# (−)-Oleocanthal Combined with Lapatinib Treatment Synergized against HER-2 Positive Breast Cancer In Vitro and In Vivo

**DOI:** 10.3390/nu11020412

**Published:** 2019-02-15

**Authors:** Abu Bakar Siddique, Hassan Y. Ebrahim, Mohamed R. Akl, Nehad M. Ayoub, Amira A. Goda, Mohamed M. Mohyeldin, Suresh K. Nagumalli, Wael M. Hananeh, Yong-Yu Liu, Sharon A. Meyer, Khalid A. El Sayed

**Affiliations:** 1 School of Basic Pharmaceutical and Toxicological Sciences, College of Pharmacy, University of Louisiana at Monroe, 1800 Bienville Drive, Monroe, LA 71201, USA; siddiqab@warhawks.ulm.edu (A.B.S.); hassanchem.phd@outlook.com (H.Y.E.); mohamedreda_pharmacy@yahoo.com (M.R.A.); amirakareem16@gmail.com (A.A.G.); mohyelmm@warhawks.ulm.edu (M.M.M.); nagumask@warhawks.ulm.edu (S.K.N.); yliu@ulm.edu (Y.-Y.L.); meyer@ulm.edu (S.A.M.); 2 Department of Clinical Pharmacy, Faculty of Pharmacy, Jordan University of Science and Technology, Irbid 22110, Jordan; nmayoub@just.edu.jo; 3 Department of Pathology and Public Health, Faculty of Veterinary Medicine, Jordan University of Science and Technology (JUST), Irbid 22110, Jordan; whananeh@just.edu.jo

**Keywords:** breast cancer, combination, HER2/neu, lapatinib, c-Met, (−)-Oleocanthal

## Abstract

Dysregulation of epidermal growth factor receptor (EGFR)/human epidermal growth factor-2 (HER2) family is a hallmark of aggressive breast cancer. Small-molecule tyrosine kinase inhibitors are among the most effective cancer targeted treatments. (−)-Oleocanthal (OC) is a naturally occurring phenolic secoiridoid lead from extra-virgin olive oil with documented anti-cancer activities via targeting mesenchymal epithelial transition factor (c-Met). Dysregulation of c-Met promotes aggressiveness to breast cancer-targeted therapies. Lapatinib (LP) is an FDA-approved dual EGFR/HER2 inhibitor for HER2-amplified breast cancer. HER2-Positive tumor cells can escape targeted therapies like LP effects by overexpressing c-Met. Combined OC-LP treatment is hypothesized to be mechanistically synergistic against HER2-overexpressing breast cancer. Combined sub-effective treatments of OC-LP resulted in synergistic anti-proliferative effects against the HER2-positive BT-474 and SK-BR-3 breast cancer cell lines, compared to OC or LP monotherapy. Antibody array and Western blot analysis showed that combined OC-LP treatment significantly inhibited EGFR, HER2, and c-Met receptor activation, as well as multiple downstream signaling proteins, compared to individual OC or LP treatment. OC-LP Combination significantly inhibited invasion and migration of breast cancer cells through reduced activation of focal adhesion kinase (FAK) and paxillin. Combined treatment of OC-10 mg/kg with LP-12.5 mg/kg suppressed more than 90% of BT-474 tumor cells growth in a nude mouse xenograft model, compared to individual OC or LP treatment. Activated c-Met, EGFR, HER2, and protein kinase B (AKT) were significantly suppressed in combination-treated mice tumors, compared to OC or LP monotherapy. This study reveals the OC future potential as combination therapy to sensitize HER2-overexpressing breast cancers and significantly reduce required doses of targeted HER family therapeutics.

## 1. Introduction

The epidermal growth factor (ErbB, HER) family consists of four transmembrane receptor tyrosine kinases (RTKs); HER1 to HER4 [1]. Their dysregulations are strongly implicated in the pathogenesis of various human malignancies, associated with poor prognostic outcomes, aggressive phenotype profile, and poor disease outcomes, including reduced overall survival [2,3,4,5]. About 20–30% of breast cancer (BC) cases are characterized by overexpression of HER2 [6,7]. The introduction of effective HER2-targeted therapies in clinical practice has dramatically improved outcomes among HER2-positive BC patients [3,6,8]. One of the clinically available HER2-targeted therapies is lapatinib (LP), an orally active small-molecule dual tyrosine kinase inhibitor (TKI) of epidermal growth factor receptor (EGFR) and HER2 kinases [8,9]. Through inhibiting receptor activation, LP suppressed downstream signaling of HER2 through major downstream proteins, such as extracellular signal-regulated kinases (ERK)1/2 and AKT [10,11]. Despite success as HER2-targeted therapy, limitations to LP treatment monotherapy involved the emergence of both primary and acquired resistance among HER2-positive BC patients and multiple off-target side effects [12,13,14]. Non-HER2-targeted therapy has also been shown to be a promising strategy of overcoming resistance and restoring the activity of HER2-targeted treatments [15].

c-Met is an RTK associated with BC development and progression [16,17]. Activation of c-Met in cancer cells by its natural ligand, the hepatocyte growth factor (HGF), accelerates proliferation, scattering, migration, invasion, and metastasis [16]. c-Met crosstalk with HER RTK members and can be a dimerization partner with HER2 [18]. Amplification of c-Met also promotes resistance to multiple anti-HER targeted therapies [18,19,20,21]. Thus, it is hypothesized that multi-targeted combination therapy using c-Met inhibitor could be appealing to enhance treatment effects of anti-HER2 targeted therapies and chemosensitize HER2-positive BC.

(−)-Oleocanthal (OC) is a natural phenolic secoiridoid from extra-virgin olive oil (EVOO) known for its anti-oxidant, anti-bacterial, and anti-inflammatory activities [22,23,24,25]. The anticancer activity of OC has been demonstrated in cell culture and animal models against different cancer types, including breast, prostate, colorectal, skin, and hepatocellular carcinoma [24,25,26]. In BC, OC was shown to effectively inhibit proliferation, migration, and invasion of BC cells both in vitro and in vivo [22,24,25,26]. Molecular modeling, cell culture, and animal studies clearly revealed c-Met as a molecular target to OC, through inhibiting its tyrosine kinase domain [27,28]. The anticancer activity of OC was associated with the suppression of multiple downstream effectors, such as the phosphoinositide-3 (PI3K)-AKT- mechanistic target of rapamycin (mTOR), protein superfamily of small guanosine triphosphatases (Ras)- Mitogen-activated protein kinase (MAPK), Janus kinase (JAK)- Signal transducer and activator of transcription 3 (STAT), and heat shock protein-90 (HSP90) signaling pathways [25,27,28,29,30,31].

Considering the crosstalk between c-Met and the HER family receptors [18,19,20], along with its involvement in mediating resistance to anti-HER targeted therapies [18,19,20,21], for the first time this study primarily aimed to investigate the in vitro and in vivo effectiveness of the combined treatments of the c-Met inhibitor EVOO-derived phenolic lead OC with the targeted therapeutic anti-EGFR/HER2 drug LP against HER2-positive BC.

## 2. Materials and Methods

### 2.1. Chemicals, Reagents, and Antibodies

All reagents purchased from Sigma-Aldrich (St. Louis, MO, USA), unless otherwise stated. All primary, secondary antibodies are purchased from Cell Signaling Technology (Beverly, MA, USA), unless otherwise stated. Hepatocyte growth factor (HGF) and epidermal growth factor (EGF) purchased from PeproTech Inc. (Rocky Hill, NJ, USA). Lapatinib (LP) purchased from AstaTech, Bristol, PA, USA.

### 2.2. Cell Lines and Culture Conditions

Human BC cell lines BT-474 and SK-BR-3, as well as the non-tumorigenic MCF-12A human mammary epithelial cells obtained from the American Type Culture Collection (ATCC, Manassas, VA, USA). Both BT-474 and SK-BR-3 are HER2-enriched BC cells and responsive to the anti-HER2 therapy trastuzumab [32]. BC cells were cultured in Roswell Park Memorial Institute (RPMI-1640) /Dulbecco’s Modified Eagle’s medium (DMEM) media supplemented with 10 % fetal bovine serum (FBS), penicillin G (100 U/mL) and streptomycin (100 ng/mL). MCF-12A cells were maintained in DMEM/F12 media supplemented with 5% horse serum, 0.5 µg/mL hydrocortisone, 20 ng/mL EGF, 100 U/mL penicillin G, 0.1 mg/mL streptomycin, and 10 µg/mL insulin. All cells were maintained in a humidified incubator at 37 °C with 5% CO_2_. For sub-culturing, cells were washed with Ca^2+^- and Mg^2+^-free phosphate-buffered saline (PBS) and incubated in 0.05% trypsin containing 0.02% Ethylenediaminetetraacetic acid (EDTA) in PBS for 5–15 min at 37 °C. 

### 2.3. Experimental Treatments 

OC was extracted from EVOO (The Governor, batch 5-214000-242017) [27,28,29] using liquid-liquid extraction process and further purification has been done using Sephadex LH-20 column purity of OC was determined using HPLC analysis on a Shimadzu high-performance liquid chromatography (HPLC) system equipped with ultra violet (UV)/Visible variable wavelength detector and a purity of >99% was confirmed [27,28,29]. Quantitative ^1^H NMR in deutrated chloroform-d_3_ (CDCl_3_) was acquired on a JEOL Eclipse ECS-400 nuclear magnetic resonance (NMR) spectrometer. OC and LP dissolved in dimethyl sulfoxide (DMSO) to provide a 25 mM stock solution. These stock solutions used to prepare various treatment concentrations. The final concentration of DMSO maintained the same in all treatment groups within a given experiment and never exceeded 0.1 %.

### 2.4. Cell Viability Assay 

Cells were seeded into a 96-well plate at a density of 1 × 10^4^ cells/well (6 replicates/group) in 10 % FBS RPMI-1640 media and allowed to attach overnight. Next day, cells were divided into different treatment groups and exposed to respective control or experimental treatments with various concentrations of either OC or LP alone or in combination for 48 h in media containing 40 ng/mL of HGF and EGF as mitogens. At the end of treatment duration, the viable cell number was determined using 3-(4,5-dimethylthiazol-2-yl)-2,5-diphenyl-tetrazolium bromide (MTT) assay [33]. MTT was added to each well at a final concentration of 1.0 mg/mL. After 4 h incubation at 37 °C, media was removed, and formazan crystals were dissolved in DMSO (100 µL/well). Optical density was measured at 570 nm on a microplate reader (BioTek, Winooski, VT, USA). The number of cells/well was calculated against a standard curve prepared by plating various numbers of cells at the start of each experiment. 

### 2.5. Western Blot Analysis

BC cells were initially plated at 1 × 10^6^ cells/100 mm culture plates in Roswell Park Memorial Institute (RPMI)-1640 media supplemented with 10% fetal bovine serum (FBS) and allowed to adhere overnight. Cells were then washed with phosphate buffered saline (PBS) and treated with the respective control or treatment media containing various concentrations of OC, LP, or their combination for 48 h. Cells were then harvested and washed twice with cold PBS, resuspended and lysed in Radioimmunoprecipitation assay (RIPA) buffer (Qiagen Sciences Inc., Valencia, CA, USA) at 4 °C for 30 min. Lysates were centrifuged for 10 min at 14,000× *g* and supernatants were stored at −80 °C as whole cell extracts. Protein concentration was determined by the Pierce BCA Protein Assay (Thermo Fisher Scientific Inc., Rockford, IL, USA). Proteins were separated on 10% sodium dodecyl sulfate polyacrylamide gel electrophoresis (SDS-PAGE) gels and transferred to polyvinylidene difluoride membranes. Membranes blocked with 2% bovine serum albumin (BSA) and incubated with the indicated primary antibodies. Corresponding horseradish peroxidase-conjugated secondary antibodies were used against each primary antibody. Proteins were detected using ChemiDoc XRS chemiluminescent gel imaging system and analyzed using Image Lab software (Bio-RAD, Hercules, CA, USA) [27,28]. Visualization of β-tubulin was used to ensure equal sample loading in each lane. Experiments were repeated three times and representative image presented in figures.

### 2.6. Cell Cycle Assay 

Cells in the various treatment groups were trypsinized and then resuspended in ice cold PBS, fixed with cold (−20 °C) 70% ethanol, and stored at 4 °C for 2 h. Afterwards, cells were rehydrated with ice cold PBS and then incubated with DNA staining buffer (sodium citrate 1 mg/mL, Triton-X-100 3 µL/mL, propidium iodide (PI) 100 µg/mL, ribonuclease A 20 µg/mL) for 30 min at 4 °C in the dark. DNA content was then analyzed using a fluorescence-activated cell sorter (FACS) Calibur flow cytometer (BD Biosciences, San Jose, CA, USA). For each sample, 10,000 events were recorded, and histograms were generated using CellQuest software (BD Biosciences, San Jose, CA, USA) [27]. All experiments were repeated at least three times.

### 2.7. Cell Apoptosis Assay

Cell apoptosis assay was conducted using Annexin V- Fluorescein isothiocyante (FITC) Early apoptosis detection kit (Cell Signaling Technology, Beverly, MA, USA). Cells in each treatment group were trypsinized and then washed twice with ice cold PBS, stained with Annexin V-FITC and PI in the binding buffer, and detected by flow cytometry (FCM) after 10 min incubation at room temperature in the dark. Dot plots were generated using CellQuest software (BD Biosciences, San Jose, CA, USA) [27].

### 2.8. Antibody Array

Explorer Antibody Microarray conducted using Full Moon Biosystems; Sunnyvale, CA, USA. Protocol is available at https://www.fullmoonbio.com/products/antibody-array/.

### 2.9. Migration and Invasion Assays

Migration and invasion of BC cells were assessed using CytoSelect 24-well Cell Migration and Invasion Assay kit (CBA-100-C, Cell Biolabs) following manufacturer instructions [27,28]. In brief, 1.5 × 10^5^ cells placed on an 8-μM pore size insert. After incubation for 24 h or 48 h, the non-migratory/non-invasive cells the upper chamber were carefully removed with cotton-tipped swabs, and the migratory/invasive cells processed per vendor’s protocol and read by a plate reader (Versamax tunable microplate reader, Molecular Devices) at λ560 nm. Before removing the cells from the upper chamber, the non-migratory cells visualized by a Nikon ECLIPSE TE200-U microscope (Nikon Instruments Inc., Melville, NY, USA). Digital images were captured using Nikon NIS Elements software (Nikon Instruments Inc., Melville, NY, USA).

### 2.10. BT-474 Nude Mice Xenograft Tumor Model 

Foxn^1nu^/Foxn^1+^ nude mice were obtained from Envigo (Indianapolis, IN, USA) and maintained with sterilized food and water. All procedures were conducted in accordance with the recommendations in the Guide for the Care and Use of Laboratory Animals [34] and approved by the University of Louisiana-Monroe Institutional Animal Care and Use Committee (IACUC, Protocol Number: 15OCT-KES-01). Five female nude mice at 4–5 weeks old, 20–23 g average weight, used for each group. Mice were anaesthetized using ketamine and xylazine mixture as previously described [27,28]. A 0.72-mg 60-day release 17β-estradiol pellet (Innovative Research) was then implanted into the interscapular region of each mouse. Every mouse was then subcutaneously injected with BT-474 cells (5 × 10^6^ cells in 30:30 µL Matrigel/RPMI-1640) under the second mammary gland fat pad [27,28]. When tumors were palpable, 30–40 mm^3^ volume average, and of the same volume at 14 days, the mice were randomized into four different groups and treated with the following treatments: (i) Vehicle control (DMSO/saline), intraperitoneal (ip), using the maximal volume used for treatments; (ii) OC 10 mg/kg, ip, 3X/week; (iii) LP 12.5 mg/kg, oral, 5X/week; (iv) OC 10 mg/kg, ip, 3X/week plus LP 12.5 mg/kg, oral, 5X/week. Mice were monitored by measuring tumor volume every third day, body weight and clinical observation. Tumor volume (V) was calculated using the formula V = (L × W^2^)/2. Percentage of tumor growth inhibition (% TGI) was measured on the last day of study for drug-treated compared with vehicle-treated mice. % TGI is calculated as 100 × {1 − [(treated final day − treated day 1)/(control final day − control day 1)]} [27,28,30].

### 2.11. Hematoxylin and Eosin Y (H&E) Staining 

Tumor sample portions were cut into small slices (3–5 mm thick), and fixed in 10% neutral buffered formalin for 48 h. The tissues were transferred to 70% ethanol, processed, and embedded in paraffin. Paraffin-embedded tumors were sliced into 5 µM sections using a Leica RM2035 microtome and mounted on positively charged slides. Paraffin sections were dewaxed in xylene, rinsed in alcohol, rehydrated in water and finally tumor slides were stained with H&E. Following the last submersion into xylene, the slides were permanently mounted using permount and a coverslip. 

### 2.12. Statistics

Values are expressed as mean ± standard error mean (S.E.M.) and analyzed using statistical package for GraphPad Prism software version 8 using One-way ANOVA followed by Tukey’s test; *p* < 0.05 were considered statistically significant. IC_50_ was determined using GraphPad Prism software version 8. Combination data were analyzed, and results showed as combination index (CI) values according to the median-effect principle, where CI < 1, =1, and >1 indicate synergism, additive effect, and antagonism, respectively. CI values calculated as follows: CI = [X_c_/X + T_c_/T], where X and T stand for the concentrations of individual combination ingredients, OC and LP, that induce 50% cell growth inhibition (IC_50_); X_c_ and T_c_ are the concentrations of combination ingredients that induce 50% cell growth inhibition when used combined as determined by non-linear regression curve fit analysis [28,35]. Dose reduction index (DRI) values for OC and LP calculated as follows: DRI (−)-Oleocanthal = OC IC_50_/OC combination IC_50_ and DRI lapatinib = LP IC_50_/LP combination IC_50_. 

## 3. Results

### 3.1. Effect of OC, LP, and Their Combined Treatments on Growth of HER2-Positive BC and Non-Tumorigenic Cells

**Figure 1:**
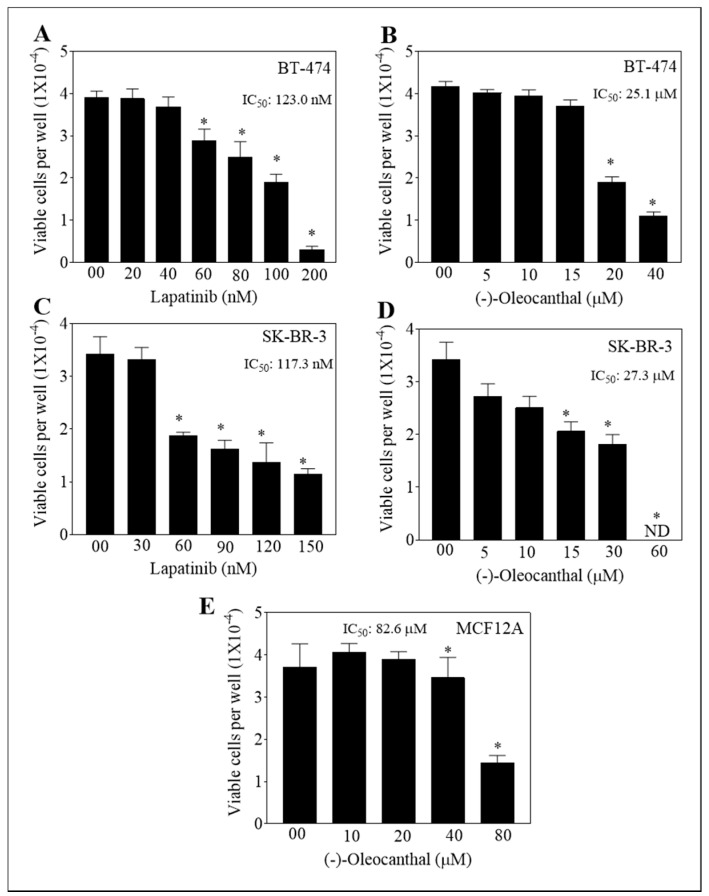
Effect of (−)-Oleocanthal (OC) and Lapatinib (LP) treatment on the growth of HER2-overexpressing BC cells. (**A**,**B**) Effect of LP or OC treatment on the growth of BT-474 cells after 48 h culture period. (**C**,**D**) Effect of LP or OC treatment the on growth of SK-BR-3 cells after 48 h culture period. (**E**) Effect of OC treatment on the growth of MCF-12A cells after 48 h culture period. In these assays, cells were plated at a density of 1 × 10^4^ cells/well in 96-well plates and maintained in media supplemented with 10 % FBS and allowed to adhere overnight. Cells then treated with vehicle-control or increasing concentrations of OC or LP in serum-free media containing hepatocyte growth factor (HGF) and Epidermar growth factor (EGF) 40 ng/mL as mitogens for 48 h. At the end of treatment, viable cell number determined by the MTT colorimetric assay. Vertical bars indicate mean cell count ± SEM (*n* = 6) in each treatment group. * *p* < 0.05 as compared with vehicle-treated controls. ND, not detectable.

The anti-proliferative activity of OC-LP was evaluated in BT-474 and SK-BR-3 cells, representing HER2-positive BC (Figure 1A–D). Monotherapy of OC or LP treatments showed a dose-dependent inhibition of growth of BT-474 and SK-BR-3 cells, with IC_50_ values of 25.1 µM, 27.3 µM, 123.0 nM and 117.3 nM, respectively. OC showed minimal effects on the viability of the non-tumorigenic mammary epithelial MCF12A cells with an IC_50_ of 82.6 µM after 48 h treatments (Figure 1E). OC was selective to malignant cells.

**Figure 2:**
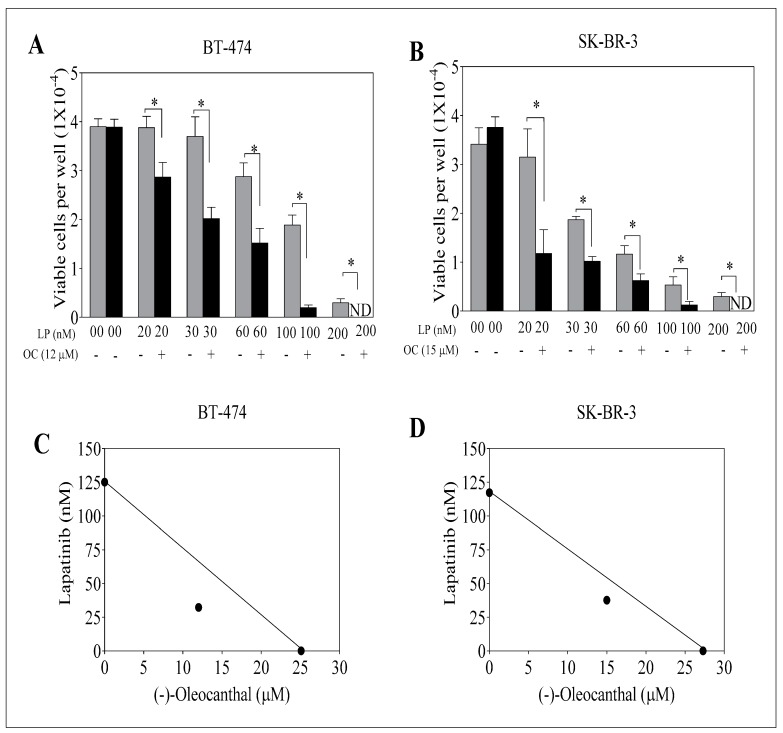
Effect of combined OC and LP treatments on growth of HER2-overexpressing BC cells. (**A**) Effect of sub-effective OC and LP combined treatment on BT-474 cells after 48 h culture period. (**B**) Effect of sub-effective OC and LP combined treatment on SK-BR-3 cells after 48 h culture period. Cells were plated at a density of 1 × 10^4^ cells/well in 96-well plates and maintained in media supplemented with 10% FBS and allowed to adhere overnight. Next day, cells were treated with vehicle-control or sub-µM dose of OC or nM dose of LP either alone or combined in serum-free media containing HGF and EGF 40 ng/mL as mitogens for 48 h. At the end of treatment, viable cell number determined by the MTT colorimetric assay. Vertical bars indicate the mean cell count ± SEM (*n* = 6) in each treatment group. * *p* < 0.05 as compared with individual LP treatment. (**C**,**D**) Isobolograms of combined OC and LP anti-proliferative effect in BT-474 and SK-BR-3 cells, respectively. IC_50_ concentrations for OC and LP plotted on the *X*- and *Y*-axis, respectively. The solid line connecting these points represents the concentration of each compound required to induce the same relative growth inhibition when used in combination if the interaction between the compounds is additive. The data point on each isobologram represents the actual concentrations of OC and LP, which induced 50% inhibition of cell growth when used in combination. ND; not detectable.

To assess the effect of combined OC and LP treatments, BT-474 and SK-BR-3 BC cells treated with sub-μM doses of OC at 12 µM or 15 µM, respectively (Figure 2A,B). Isobologram analysis of the effect of OC and LP combination treatments in both cell lines indicated synergistic inhibition of cell growth (Figure 2C,D). Calculated combination index (CI) value for the combination treatments of OC-LP indicated synergism, with values less than 1 in both BT-474 and SK-BR-3 cells (Supplementary Table S1). In BT-474 cells, the CI value was 0.74 for a combined 30 nM LP and 12 µM OC treatment. In SK-BR-3 cells the CI value was 0.87 for 60 nM LP and 15 µM OC combined treatment. In addition, the dose reduction index (DRI) values for combined OC-LP treatments showed multiple-fold reductions of both compounds (Supplementary Table S1). After determining the OC and LP interaction levels and the concentrations resulting in synergism, subsequent experiments were conducted using OC 12 µM with 30 nM LP in BT-474 cells and OC 15 µM with 60 nM LP in SK-BR-3 cells.

### 3.2. Effect of Combined OC and LP Treatment on Target RTKs and Downstream Effectors in HER2-Positive BC Cells 

**Figure 3:**
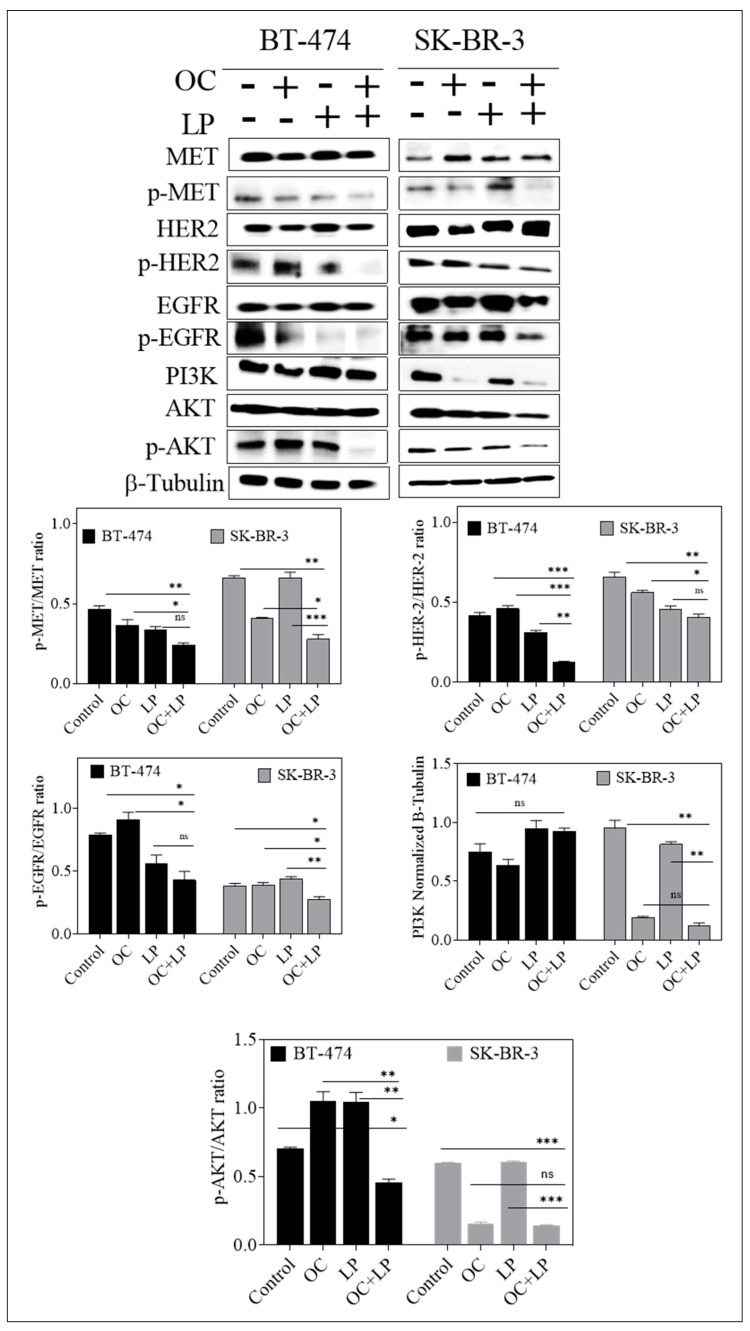
Effect of combined OC and LP treatment on target RTKs and downstream effectors and proliferation markers in HER2-positive BC cells. Western blot analysis for the effect of combined OC and LP treatment on RTKs, and PI3K/AKT signaling pathways. Cells were plated at 1 × 10^6^ cells/100 mm culture plates in RPMI-1640 media supplemented with 10% FBS and allowed to adhere overnight. Next day, cells incubated with respective OC, LP, or combined treatment in serum-free media containing HGF and EGF 40 ng/mL as mitogens for 48 h. At the end of the treatment period, the whole cell lysates were prepared then subjected to polyacrylamide gel electrophoresis and Western blot analysis. β-Tubulin was visualized to ensure equal sample loading in each lane. Image Lab densitometric analysis performed on all blots and the integrated optical density of each band was normalized with the corresponding β-tubulin, as shown under their respective Western blot images. * *p* < 0.05, *** *p* < 0.001 as compared with either individual OC or LP treatment or vehicle treatment.

To investigate the effects of OC-LP combination on the total and active levels of MET, HER2, and EGFR RTKs, Western blot analysis was performed. Both BC cell lines treated with individual or combined OC and LP for 48 h. Combined OC-LP treatment significantly suppressed the activation of c-Met, HER2, and EGFR in both cell lines as indicated by reduced receptor phosphorylation levels compared to individual OC or LP treatment (Figure 3). However, in BT-474 cells combination treatment slightly reduced the activated levels of c-Met, HER2, and EGFR, which may be because of OC individual effect since it also reduced the total levels of c-Met, HER2, and EGFR (Figure 3). In addition, BT-474 cells combined OC-LP treatment did not affect the PI3K downstream signaling but significantly suppressed the activation of AKT (Figure 3), compared to individual OC or LP treatment. In SK-BR-3 cells combination treatment reduced total c-Met and EGFR but eventually there is no effect of LP on p-Met reduction. Although, OC individual and combination treatment reduced PI3K downstream signaling protein, but combination treatment significantly suppressed the activation of AKT as compared to individual OC or LP treatment (Figure 3).

### 3.3. Effect of Combined OC and LP Treatment on Cell Cycle Progression in HER2-Positive BC Cells

To further investigate the molecular mechanisms associated with growth suppression observed with combined OC-LP treatment, cell cycle analysis was assessed by flow cytometry applying propidium iodide (PI) staining (Supplementary Figure S1A). Cell cycle analysis demonstrated an increase in the proportion of BT-474 cancer cells in G1 phase in combined OC-LP treatment (58%), compared to G1 cells in the vehicle control treatment (53%, Supplementary Figure S1B). However, the impact of combined OC-LP treatment on the G1 cell population was less remarkable in SK-BR-3 cells (Supplementary Figure S1B). Supplementary Figure S1C shows the effect of OC-LP combination treatments on cell cycle key regulatory molecules. Western blot analysis showed that combined OC-LP treatment resulted in downregulation of cyclins D1 and D3, as well as reduced total levels of cyclin-dependent kinase-6 (CDK-6) in both BT-474 and SK-BR-3 cells (Supplementary Figure S1C). In addition, the combination was associated with increased total levels of cell cycle arrest proteins p21, also known as p21^WAF1/Cip1^, and p27 in treated cancer cells, compared to their respective vehicle-treated control groups (Supplementary Figure S1C). 

### 3.4. Pro-Apoptotic Effects of Combined OC-LP Treatment in HER2-Positive BC Cells 

**Figure 4:**
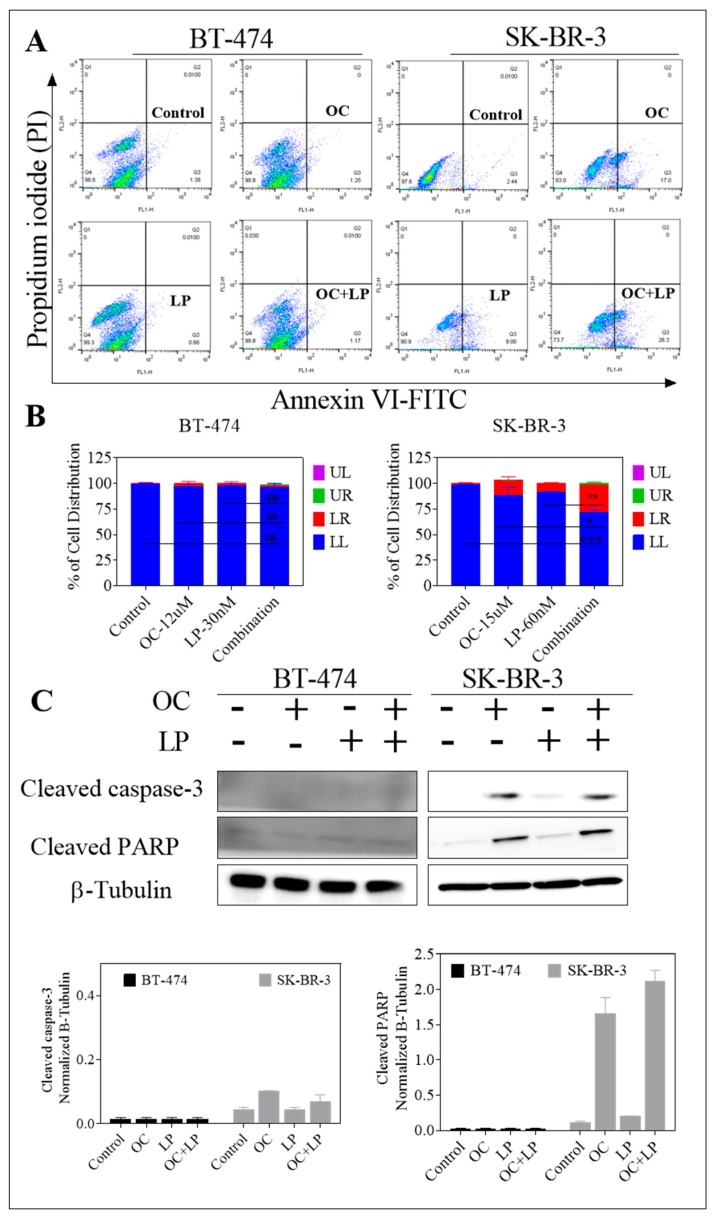
Pro-apoptotic effects of combined OC-LP treatment in HER2-positive BC cells. (**A**) Flow cytometry analysis for OC combined with LP treatment in BT-474 and SK-BR-3 BC cells. Cells were plated at a density of 5 × 10^6^ cells/100 mm culture plates, allowed to attach overnight. Afterwards, cells were incubated in the respective control, OC, LP, or LP-OC combination in RPMI-1640 medium containing 40 ng/mL HGF and EGF for 24 h. At the end of the experiment, cells in each treatment group were trypsinized, washed then resuspended in ice-cold 1X Annexin V binding buffer. Afterwards, cells treated as described earlier. In the dot plot of double variable flow cytometry, LL quadrant (FITC^−^/PI^−^) shows living cells; LR quadrant (FITC^+^/PI^−^) represents early apoptotic cells; UR quadrant (FITC^+^/PI^+^) stands for late apoptotic cells and UL quadrant (FITC^+^/PI^+^) stands for necrotic cells. (**B**) Western blot analysis of cleaved caspase 3 and cleaved PARP performed after 24 h incubation. Cells analyzed to examine cell death by measuring cleaved caspase 3 and cleaved PARP detected by Western blotting. In all the above experiments, whole cell lysates were prepared for subsequent separation by polyacrylamide gel electrophoresis followed by Western blot analysis. Imaging and analysis performed as described earlier. * *p* < 0.05, *** *p* < 0.001 as compared with either individual OC or LP treatment or vehicle treatment.

The cytotoxic activity of combined OC-LP treatment was assessed using the apoptotic marker Annexin V and the oncotic marker PI in treated breast cancer cells applying flow cytometry (Figure 4A). In BT-474 cells, treatment with OC (12 µM) (1.25%), LP (30 nM) (0.68%), or the combination treatment (1.17%) did not increase Annexin V labeling among treatment groups as compared to control cells (1.38%, Figure 4B), which indicated cytostatic rather than cytotoxic effect. Meanwhile, combined treatment of OC (15 µM) with LP (60 nM) also increased the proportion of SK-BR-3 cells with positive Annexin V staining (26.3%), compared to the vehicle-treated control cells (2.44%, Figure 4B). Surprisingly, OC individual treatment induced significant early apoptosis (17%), compared to LP treatment (9.08%) in SK-BR-3 cells. Furthermore, Western blot analysis further revealed the apoptotic effect in treated SK-BR-3 cells as indicated by increased cleaved caspase 3 and cleaved Poly (ADP-ribose) polymerase (PARP) expressions with either OC individual or combined OC-LP treatments (Figure 4C). Apoptosis markers were not detected in BT-474 cells treated with either individual OC or LP or combined, compared to the vehicle control-treated cells as indicated by lack of cleaved caspase 3 and cleaved PARP (Figure 4C).

### 3.5. Effect of Combined OC-LP Treatment on Cell Protein Array Assay in BT-474 BC Cells 

A high-throughput ELISA-based antibody microarray was performed to identify candidate proteins and signaling pathways associated with the molecular effects of combined OC-LP treatment in BT-474 cells (Supplementary Figure S2). Microarray analysis data demonstrated that combination treatment suppressed phosphorylation of multiple molecular targets, including HER-2, FAK, JAK1 and MEK2 (Supplementary Figure S2). Alternatively, the combined OC-LP treatment exerted little effect on the phosphorylation of IGF-1R in treated BT-474 cells (Supplementary Figure S2).

### 3.6. Effect of Combined OC-LP Treatment on Migration and Invasion of HER2-Positive BC Cells and Downstream Signaling Effectors 

**Figure 5:**
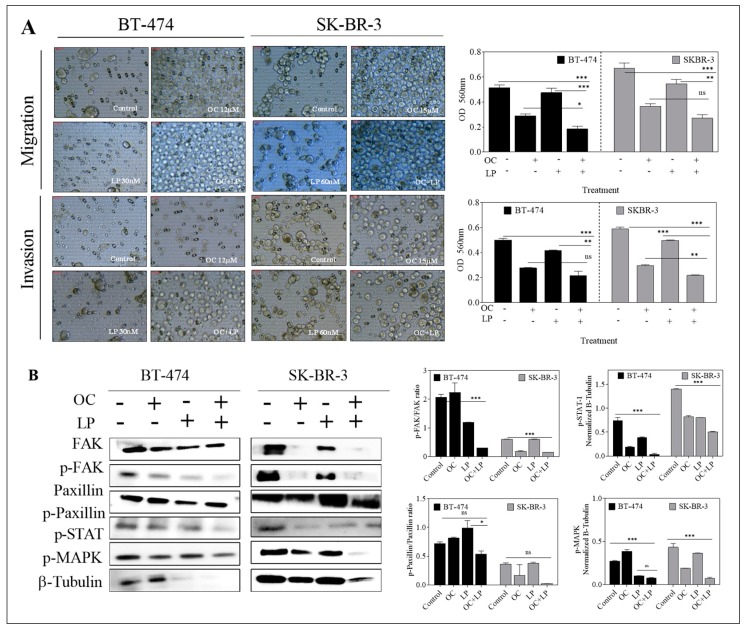
Effect of combined OC and LP treatment on migration and invasion of HER2-positive BC cells and downstream signaling effectors. (**A**) Effect of OC and LP treatment on migration and invasion of BT-474 and SK-BR-3 BC cells. Optical density in migration and invasion assay in BT-474 and SK-BR-3 cells. Cells were seeded according to the manufacturer protocol and as described in “Methods”. In both assays, the provided images were taken for the upper surface of the inserts (**B**) Western blot analysis for the effect of combined OC and LP treatment on total and active levels of proteins controlling cell migration and invasion. Whole cell lysates were prepared for separation by polyacrylamide gel electrophoresis followed by Western blot analysis. Imaging and analysis were performed as mentioned earlier. * *p* < 0.05, *** *p* < 0.001 as compared with either individual OC or LP treatment or vehicle treatment. ns, not statistically significant.

Combined OC-LP treatment inhibited the migration and invasion of both HER2-positive breast cancer cell lines, compared to vehicle-treated control cells, as well as OC or LP individual treatments (Figure 5). Western blot analysis further demonstrated molecular pathways significantly altered by the anti-migratory and anti-invasive effects of combination treatment in cancer cells in compared to OC or LP individual treatment. In both BT-474 and SK-BR-3 cells, combined OC-LP treatments resulted in reduced both total, as well as phosphorylated levels of FAK, compared to vehicle control-treated cells (Figure 5). Individual OC treatment also reduced the total level of FAK in SK-BR-3 cells while the suppression of paxillin and its phosphorylated form was more evident for combination treatment on both SK-BR-3 cells and BT-474 cells (Figure 5). Multiple mitogenic signaling pathways significantly suppressed by combined OC-LP treatment, including the STAT and mitogen-activated protein kinase (MAPK) in both BT-474 and SK-BR-3 cells, compared to individual OC or LP treatment (Figure 5).

### 3.7. Effects of Combined OC-LP in BT-474 Tumor Xenografts in Nude Mouse Model

**Figure 6:**
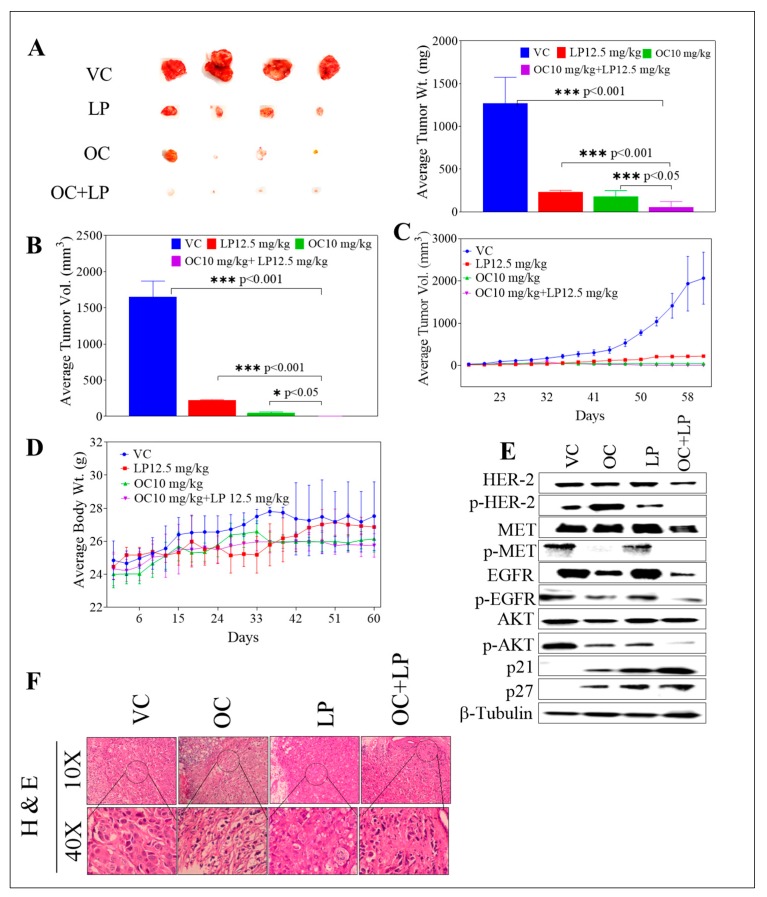
Effect of OC-LP combination treatment on tumor growth in BT-474 BC cells xenograft model. (**A**) Left panel. Representative isolated mice tumors of each experimental group collected at end of the experiment. Top row is the vehicle-treated control group, then the 12.5 mg/kg LP, oral, 5X/week, the 10 mg/kg OC, ip, 3X/week, and finally the bottom row represents the combined 10 mg/kg OC, ip, 3X/week −12.5 mg/kg LP, oral, 5X/week-treated group. Right panel. The mean tumor weight at the end of the experiment vertical bars graph. (**B**) The mean tumor volume at the end of the experiment vertical bars graph. The tumor volume (V) was calculated as V = (L × W^2^)/2, where L was the length and W was the width of tumors. Bars ± SEM. *** *p* < 0.001 as compared with either individual OC or LP treatment or vehicle treatments. (**C**) Tumor volumes monitoring over the course of the experiment. Points represent the mean tumor volume of several tumors (*n* = 5) in each experimental group over the treatment period. Error bars indicate SEM for *n* = 5. (**D**) Body weight monitoring of mice over the experiment course. Points represent mean body weight for animals in each group (*n* = 5) over the experiment duration. Error bars indicate SEM for *n* = 5. (**E**) Western blot analysis of various experimental groups for the total and active levels of study target RTKs and their downstream signaling proteins. (**F**) H&E staining of tumor samples in different study groups. Human BC cells BT-474 cultured and suspended in serum-free RPMI medium with (30:30) µL Matrigel. Cell suspensions (5 × 10^6^ cells/60 µL) subcutaneously inoculated into the second mammary gland fat pad just beneath the nipple of each athymic nude mouse to generate orthotropic breast tumor xenografts. One day before tumor cells inoculation, a 0.72-mg 60-day release 17β-estradiol pellet (Innovative Research) surgically implanted into the interscapular region of each mouse. Once the tumor is palpable, ~35 mm^3^ volume around the 14th day, mice were randomly divided into four groups, *n* = 5 each: (i) The vehicle-treated control group; (ii) the 10.0 mg/kg OC-treated group; (iii) the 12.5 mg/kg LP-treated group; and (iv) the OC 10.0 mg/kg plus LP 12.5 mg/kg—treated group. Oral treatments, vehicle control (DMSO/saline), OC, LP, and OC-LP combination, started 14 days post-inoculation and continued until the 60th day.

The anti-tumor effect of combined OC-LP treatment was investigated in vivo in an orthotopic xenograft tumor model of BT-474 cells in nude mice. The mean tumor weight for the vehicle-treated control group was 1490.32 ± 273.10 mg at the end of treatment duration. The mean tumor weight for the 10.0 mg/kg OC-treated group was 191.86 ± 40.92 mg. The mean tumor volume was 1248.52 ± 274.73, and 74.19 ± 9.47 mm^3^ for vehicle control and 10.0 mg/kg OC-treated mice groups, respectively (Figure 6A). The OC 10.0 mg/kg treatments resulted in >90% tumor growth reduction compared to vehicle-treated control animals. The mean tumor weight for the 12.5 mg/kg LP-treated group was 228.42 ± 19.85 mg and the mean tumor volume for the same treatment was 230.73 ± 8.83 mm^3^. LP treatment at 12.5 mg/kg resulted in 81.21% tumor growth reduction, compared to vehicle control-treated mice. 

Interestingly, co-administration of 12.5 mg/kg LP with 10 mg/kg OC resulted in the greatest tumor growth inhibition, compared to the vehicle and monotherapy controls. Post-hoc analysis showed a significant difference in the mean tumor weight or volume between the monotherapy OC or LP and the combination-treated group. Combined 12.5 mg/kg LP with 10 mg/kg OC-treated group resulted in a mean tumor weight of 51.25 ± 8.05 mg (Figure 6A) and mean tumor volume of 4.87 ± 0.44 mm^3^ (Figure 6B). Combined treatment resulted in over 99.0% tumor growth inhibition, compared to vehicle control-treated mice. All treatments did not cause gross changes in the body weight of the treated animals (Figure 6D). Western blot analysis of isolated mice tumors collected at the experiment end showed the downregulation of the total levels of HER2, EGFR, and c-Met in LP-OC combination-treated group. Combination treatment also caused significant downregulation of the p-HER2, p-EGFR, and p-c-Met active levels in comparison to the OC or LP individual treatments (Figure 6E and Supplementary Figure S3). Further, Western blot of mice tumors also revealed increased levels of the arrest proteins p27 and p21 for the combination-treated group, which was associated with a reduced phosphorylated active level of AKT (Figure 6E and Supplementary Figure S3). 

Histopathological analysis of various group tumors revealed the lack of defined capsule in tumors from untreated control tumor cells (Figure 6F). Mice tumors treated with the individual 10 mg/kg OC were encapsulated and tumor cells presented small, pyknotic nuclei and clear cytoplasm (Figure 6F). Occasional focal areas of necrosis were also observed. Mitotic cells were infrequent. LP 12.5 mg/kg treatment showed tumors with highly pleomorphic cells, frequent large lipid vacuoles and mitotic figures dispersed throughout the tumor section (Figure 6F). Large cells with chromosome fragmentation and apoptotic cells were also frequently observed. Mice treated with combined OC 10 mg/kg plus LP 12.5 mg/kg showed small nests of tumor cells within the connective tissue stroma. Tumor cells showed pyknotic nuclei, evident apoptotic cells, and rare mitosis (Figure 6F).

## 4. Discussion

Overexpression of HER2 protein is associated with aggressive tumor profile and poor clinical outcomes among BC patients diagnosed with HER2-positive phenotype [36,37]. Approximately 20% of human BC cases are HER2-amplified [36,37]. Multiple HER2-targeted agents have been already approved and in clinical practice for HER2-positive malignancies. Lapatinib (LP) is a small-molecule dual inhibitor of both EGFR and HER2 [9,10,11]. 

HGF/c-Met axis has been shown to strongly contribute to the development and progression of BC [17,38,39,40]. c-Met/HGF axis is dysregulated in 20–30% of clinical BC cases and is a valid and independent predictor of poor patient prognosis [39,40]. On the molecular level, the crosstalk between c-Met and HER family has been extensively studied [19,20,21]. c-Met can form heterodimers with EGFR, HER2, and HER3 in lung cancer cells [41]. These heterodimers have differential roles in tumor development in lung cancer with c-Met amplification [41]. In addition, ligand-dependent hetero-dimerization of HER family and c-Met has been shown to enhance activation of multiple downstream signaling pathways, including Src, PI3K-AkKT-mTOR, and Ras-MAPK [42,43].

Dysregulation of HGF/c-Met axis promotes acquired resistance to cancer cells treated with HER family targeted therapies [20,21,22,43,44]. Therefore, despite the proven clinical benefits of LP, multiple cancer cell phenotypes commonly escape its therapeutic effects through the activation of alternative survival pathways, including HGF/c-Met pathway [20,36,44,45,46,47,48]. Extended LP use can even induce cancer cell proliferation by promoting atypical multi-HER family heterodimerization [49]. Therefore, combining a c-Met inhibitor with LP provides a promising approach to chemosensitize the HER2-positive tumor cells and reduce resistance emergence through c-Met pathway activation [36,44,50]. 

The results of this study demonstrated the synergistic effect of combined treatment of LP with the EVOO phenolic OC against wild type HER-2-positive BC cells. Because of its well-documented activity in inhibiting c-Met activation [27], OC combined with LP in this study to further investigate possible improved therapeutic effects of this novel combination against the HER2-positive BC cells. OC-LP combination resulted in synergistic growth inhibition of the HER-2 positive BC cell lines BT-474 and SK-BR-3. Both cell lines have been shown to co-express HER2 and c-Met and were sensitive to targeted inhibitors of these receptors. Growth inhibition was associated with reduced activation of HER2, EGFR, and c-Met (Figure 3). The suppression of RTKs activation observed with combined OC-LP resulted in subsequent inhibition of multiple downstream survival and mitogenic signaling pathways in HER2-positive cells, including the PI3K, MAPK, and STAT pathways (Figure 5). In addition, the anti-proliferative activity of the combined OC-LP treatment was mediated, in-part, by the induction of G1 cell cycle arrest in cancer cells as indicated by upregulation of arrest proteins p21 and p27 and reduced levels of cyclins and cyclin D kinases (CDKs, Supplementary Figure S1). Of specific interest is the significant OC-LP combined treatment enhancement for the p21 level, which is a p53-independent master effector of multiple tumor suppressor pathways, promoting and justifying the synergistic anti-proliferative effects especially in HER2/estrogen-positive BC [51].

The synergistic interaction between OC and LP in this study enhanced cancer cell sensitivity to LP treatment evidenced by multi-fold reductions for LP concentration when combined with a sub-effective dose of OC. Combining OC with LP reduced its required IC_50_ dose to nearly 1/4th or 1/3rd of its original cytotoxic dose against BT-474 and SK-BR-3 cells, respectively (Supplementary Table S1). Isobologram analysis of the effect of OC-LP combination treatments in both cell lines indicated synergistic inhibition of cell growth (Figure 2). Combined OC-LP treatments induced apoptosis in SK-BR-3 cells as determined by detectable levels of cleaved caspase 3 and cleaved PARP in treated cells, compared to vehicle-controls (Figure 4C) while combined treatment in BT-474 cell showed a clear cytostatic effect, as indicated by the lack of the cleaved caspase 3 and cleaved PARP in treated cells. BC cell lines used in this study have different molecular profiles, doubling times, and expression of alternative growth signaling pathways. BT-474 cells represent luminal B type, estrogen, progesterone, and HER2 positive [32]. SK-BR-3 cells are positive to HER2, but negative to both estrogen and progesterone receptors [32]. 

OC-LP combination treatment suppressed ligand (HGF and EGF)-induced migration and invasion of HER2-positive BC cells. The anti-migratory and anti-invasive activities for the OC-LP combination were primarily mediated by reduced FAK and paxillin levels and activation in both BT-474 and SK-BR-3 cells (Figure 5), compared to individual OC or LP treatment. These findings are of particular significance because migration and invasion are critical steps for subsequent tumor cell metastasis, an event associated with high cancer-related mortality [17,37].

Earlier clinical data demonstrated a greater benefit of LP treatment among HER2-amplified BC patients and hormone receptor negative status, compared to those who were hormone receptor positive [45]. In line with this, this study in vitro data indicated monotherapy decreased the sensitivity of BT-474 cells to LP as a result of expression of hormone receptors [45], meanwhile combination treatment showed significant sensitivity as compared to control and individual treatment. This may be due to earlier proven ability of OC to show anti-estrogenic and ER expression reducing effects, in vitro and in vivo [28].

Combined OC (10 mg/kg)-LP (12.5 mg/kg) remarkably suppressed BT-474 tumor growth in a xenograft mouse model (Figure 6). The combined treatment lacked preliminary systemic toxicity as indicated by the sustained normal body weight of treated mice groups (Figure 6D). The significant tumor growth inhibition in combination-treated mice was mediated by reduced total and active levels of HER2, EGFR, and c-Met, compared to individual OC or LP treatments (Figure 6E), suggesting effective tumor cell sensitization. Histopathological examination of the tumor sections collected from combination treatment mice group showed significant lack of mitotic tumor cells, compared to vehicle control and individual OC and LP-treated mice, which further validate the in vivo OC-LP combination synergy and efficacy (Figure 6F). 

## 5. Conclusions

Combination of OC as a c-Met inhibitor with the dual HER2/EGFR inhibitor LP induced synergistic growth inhibition for the mitogen-mediated growth of HER2-positive breast cancer cells in vitro and in vivo. Thus, OC appears as an appealing c-Met lead natural product inhibitor that could boost the activity of the HER-targeting therapies, represented by LP, at concentrations that had no remarkable effects on the viability of non-tumorigenic cells (Figure 1). Mechanistically, introducing OC as a c-Met inhibitor in combination with LP treatment would allow the use of reduced doses of targeted therapies like LP, which would reduce future resistance emergence and drug toxicity while maintaining maximal therapeutic activity. Meanwhile, to reach the therapeutic dose of 10 mg/kg in humans, it would be required to consume every day about 700 mL of the best quality EVOO, averaging OC natural occurrence of 1 g/L, highlighting the future need to use formulated pure OC as a dietary supplement for therapeutic applications. 

## Supplementary Materials

The following are available online at http://www.mdpi.com/2072-6643/11/2/412/s1. Table S1: Combination index and dose reduction index values for OC-LP combined treatment, Figure S1. Flow cytometry analysis for cell cycle progression, Figure S2. Effect of combined OC and LP treatment on cell protein array assay, Figure S3. Densitometric quantification of pHER-2, p-Met, p-EGFR, and others.

## Abbreviations

OC	(−)-Oleocanthal
LP	Lapatinib
c-Met	Mesenchymal-epithelial transition factor
EGFR	Epidermal growth factor receptor
HER	Human epidermal growth factor receptor
FAK	Focal Adhesion Kinase
AKT	Protein kinase B
PI3K	Phosphoinositide 3-kinase
MAPK	Mitogen-activated protein kinase
JAK	Janus kinase
STAT-3	Signal transducer and activator of transcription 3
HSP90	Heat shock protein 90
RPMI	Roswell Park Memorial Institute
FBS	Fetal bovine serum
DMEM	Dulbecco’s Modified Eagle’s medium
EDTA	Ethylenediaminetetraacetic acid
NMR	Nuclear magnetic resonance
DMSO	Dimethyl sulfoxide
MTT	3-(4,5-dimethylthiazol-2-yl)-2,5-diphenyl-tetrazolium bromide
BC	Breast cancer
BCA	Bicinchoninic acid assay
BSA	Bovine serum albumin
DNA	Deoxyribonucleic acid
FITC	Fluorescein isothiocyante
CDK-6	Cyclin-dependent kinase-6
PARP	Poly (ADP-ribose) polymerase
CI	Combination index
DRI	Dose reduction index
EGF	Epidermal growth factor
ELISA	Enzyme-linked immunosorbent assay
EVOO	Extra-virgin olive oil
HGF	Hepatocyte growth factor
HPLC	High performance liquid chromatography
mTOR	Mechanistic target of rapamycin
PBS	Phosphate buffered saline
PI	Propidium iodide
TKIs	Tyrosine kinase inhibitors
UV	Ultraviolet
