# Supplementary material for: (−)-Oleocanthal Combined with Lapatinib Treatment Synergized against HER-2 Positive Breast Cancer In Vitro and In Vivo
Source: Nutrients. 2019 Feb 15;11(2):412. doi: 10.3390/nu11020412 (PMC6412724; doi:10.3390/nu11020412)
Supplement: Supplementary file 1 [file nutrients-11-00412-s001.pdf]

nutrients

Article-Supplementary

(−)-Oleocanthal Combined with Lapatinib Treatment Synergized against HER-2 Positive Breast Cancer In Vitro and In Vivo

Abu Bakar Siddique [1], Hassan Y. Ebrahim [1], Mohamed R. Akl [1], Nehad M. Ayoub [2], Amira A. Goda [1], Mohamed M. Mohyeldin [1], Suresh K. Nagumalli [1], Wael M. Hananeh [3], Yong-Yu Liu [1], Sharon A. Meyer [1] and Khalid A. El Sayed [1,*]

[1] School of Basic Pharmaceutical and Toxicological Sciences, College of Pharmacy, University of Louisiana at Monroe, 1800 Bienville Drive, Monroe, LA 71201, USA; siddiqab@warhawks.ulm.edu (A.B.S.); hassanchem.phd@outlook.com (H.Y.E.); mohamedreda_pharmacy@yahoo.com (M.R.A.); amirakareem16@gmail.com (A.A.G.); mohyelmm@warhawks.ulm.edu (M.M.M.); nagumask@warhawks.ulm.edu (S.K.N.); yliu@ulm.edu (Y.-Y.L.); meyer@ulm.edu (S.A.M.)

[2] Department of Clinical Pharmacy, Faculty of Pharmacy, Jordan University of Science and Technology, Irbid 22110, Jordan; nmayoub@just.edu.jo

[3] Department of Pathology and Public Health, Faculty of Veterinary Medicine, Jordan University of Science and Technology (JUST), Irbid 22110, Jordan; whananeh@just.edu.jo

* Correspondence: elsayed@ulm.edu; Tel.: +1-318-342-1725

Table of Contents

	Page
**Table S1**: Combination index and dose reduction index values for OC_LP combined treatment	3
**Figure S1.** Flow cytometry analysis for cell cycle progression.	4
**Figure S2.** Effect of combined OC and LP treatment on cell protein array assay.	5
**Figure S3.** Densitometric quantification of pHER-2, p-Met, p-EGFR, and others.	6

Supplementary Table S1: Combination index (CI) and dose reduction index (DRI) values for combined treatments of (-)-oleocanthal and lapatinib resulting in 50% reduction in growth of HER2-positive breast cancer cell lines.

	Monotherapy		Combination				
Cancer Cell Line	IC_{50} OC (μM)	IC_{50} LP (nM)	IC_{50} OC (μM)	IC_{50} LP (nM)	CI	DRI=OC	DRI=LP
BT-474	25.1	123	12	32.3	0.74	2.1	3.8
SK-BR-3	27.29	117.25	15	37.70	0.87	1.82	3.11

CI; combination index, DRI; dose reduction index, LP; lapatinib, OC; oleocanthal

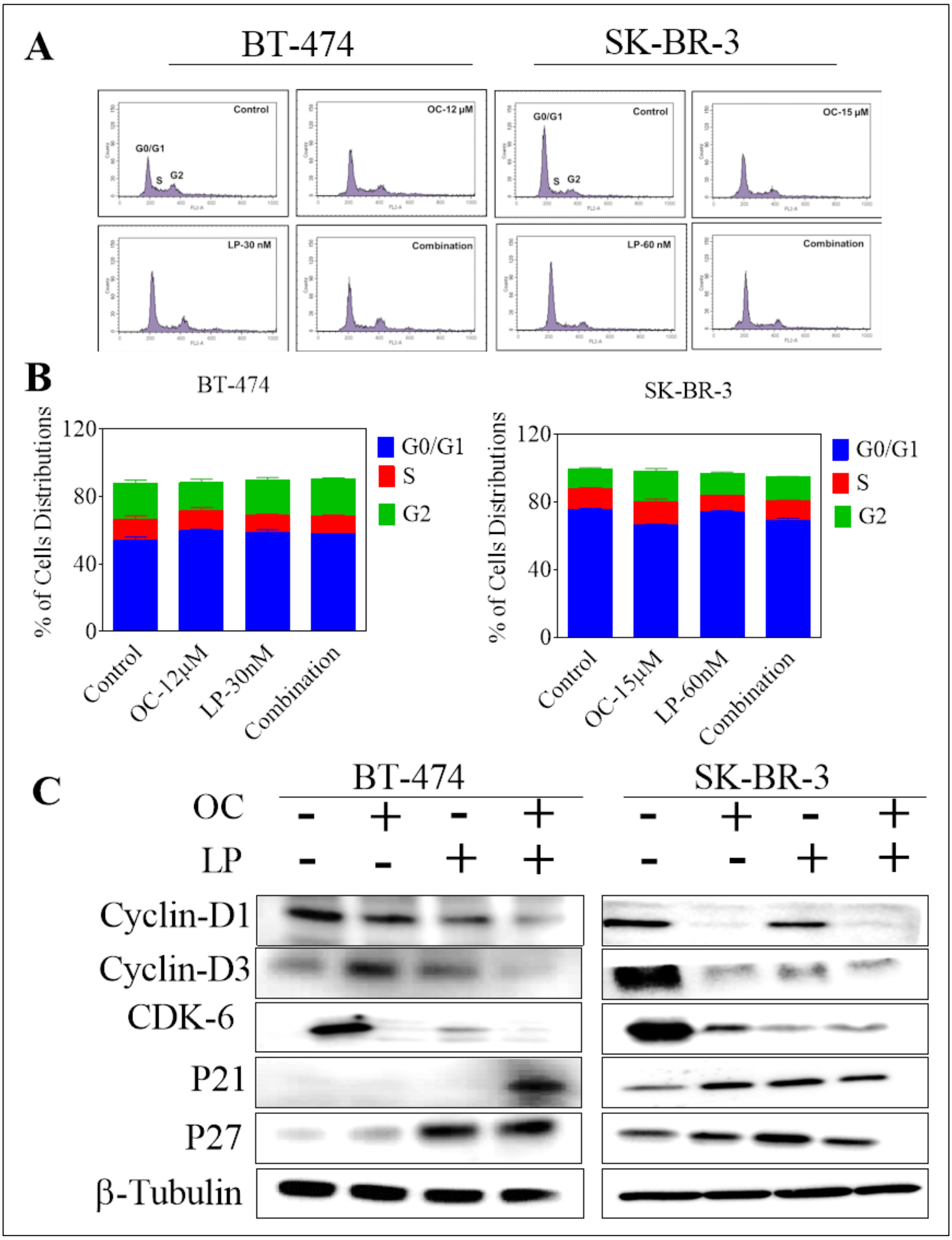

Supplementary Figure S1. (A) Flow cytometry analysis for cell cycle progression in control, LP, OC and LP-OC treatment in B-T474 and SK-BR-3 cells. Cells in the various treatment groups were synchronized in G1 phase. Briefly, BT-474 and SK-BR-3 cells were plated at a density of 1×10⁶ cells/100 mm plates in RPMI-1640 media supplemented with 10% FBS and allowed to adhere overnight. Cells were then washed twice with PBS and starved in control or treatment serum-free medium for 48 h to synchronize the cells in G1 phase. Afterwards, cells were fed various doses of OC or LP or combination as previously mentioned in serum-free defined media containing 40 ng/ml HGF and EGF as the mitogen for 24 h. Histograms generated using Cell Quest software (PI staining). (B) Histogram representing percentage distribution of cancer cells in the different phases of cell cycle in BT-474 and SK-BR-3 BC cells. (C) Western blot analysis showing the effect of previously mentioned treatments on G1/S cell cycle regulatory proteins. Cells in the various treatment groups were synchronized in G1 phase in the same way described above. Afterwards, whole cell lysates were prepared for subsequent separation by polyacrylamide gel electrophoresis followed by Western blot analysis. Imaging and analysis performed as previously mentioned.

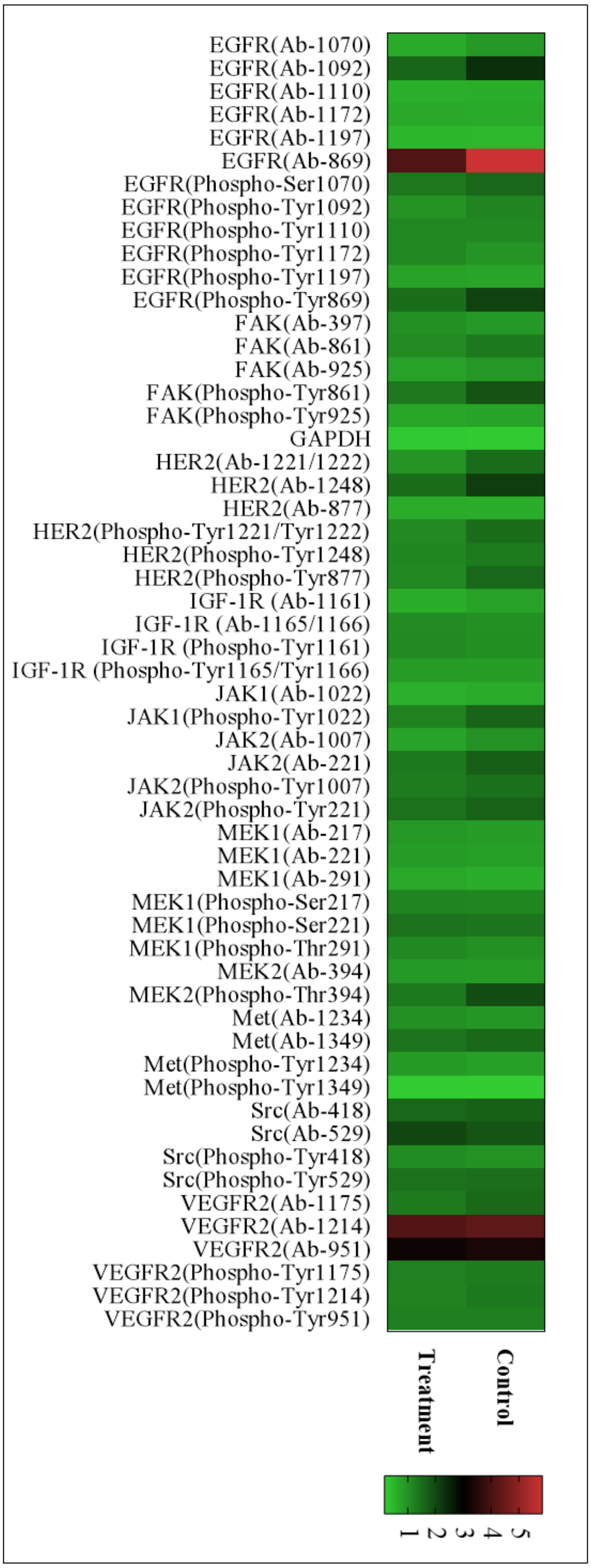

Supplementary Figure S2. Effect of combined OC and LP treatment on cell protein array assay in BT-474 BC cells.OC combined with LP treatment compared with vehicle treated sample in BT-474 breast cancer cells using antibody array assay. The whole experiment conducted according to manufacturer protocol. In general, a fold change is considered significant when the value is less than 0.5 or greater than 2. A value of 0.5 indicates that the protein amount has decreased by 50%, and a value of 2 means the protein amount has doubled. However, the cut-off value for significant fold change can vary with sample type, treatment method, dosage, and other aspects of the experiment.

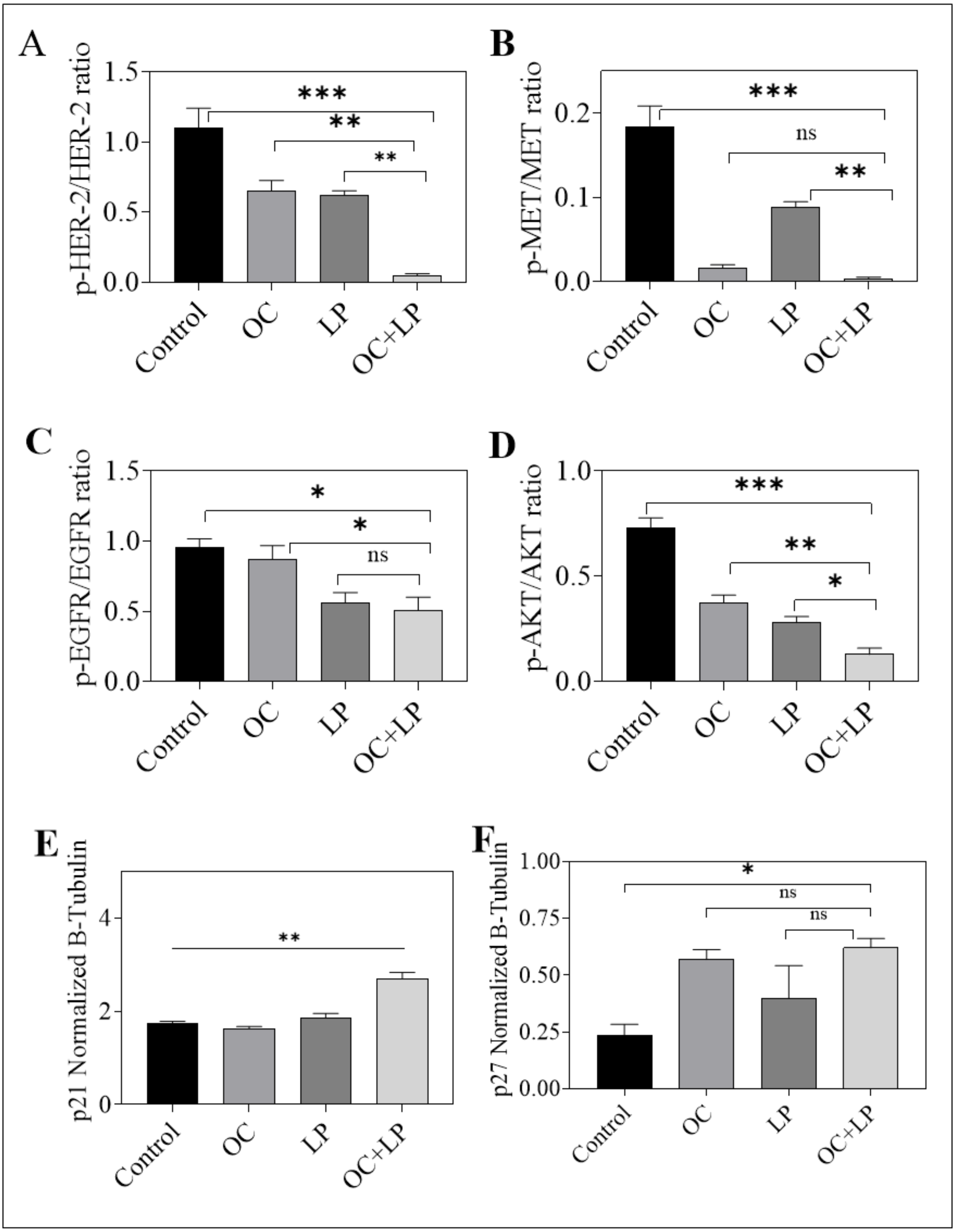

Supplementary Figure S3. Densitometric quantification of (A) pHER-2, (B) p-Met, (C) p-EGFR, (D) p-AKT, (E) p21 and (F) p27 in Western blot of mice BT-474 tumor samples. **p* <0.005, ****p* <0.001,   considered as significant. ns, not statistically significant.
